# Causes of Acute Peritonitis and Its Complication

**DOI:** 10.7759/cureus.15301

**Published:** 2021-05-28

**Authors:** Danesh Kumar, Ishan Garg, Atif Hussain Sarwar, Love Kumar, Vikash Kumar, Sonam Ramrakhia, Sidra Naz, Amna Jamil, Zoya Qamar Iqbal, Besham Kumar

**Affiliations:** 1 Internal Medicine, Liaquat University of Medical and Health Sciences, Jamshoro, PAK; 2 Clinical Medicine, Ross University School of Medicine, Bridgetown, BRB; 3 Internal Medicine, Ghulam Muhammad Mahar Medical College, Sukkur, PAK; 4 Internal Medicine, Jinnah Sindh Medical University, Karachi, PAK; 5 Medicine, Liaquat University of Medical and Health Sciences, Jamshoro, PAK; 6 Medicine, Mustafai Trust Central Hospital, Sukkur, PAK; 7 Internal Medicine, University of Health Sciences, Lahore, PAK; 8 Obstetrics and Gynecology, Jinnah Postgraduate Medical Centre, Karachi, PAK; 9 Internal Medicine, Jinnah Postgraduate Medical Centre, Karachi, PAK

**Keywords:** peritonitis, etiology, complications, typhoid ileal perforation, ruptured appendix

## Abstract

Introduction

Peritonitis is a significant cause of morbidity and mortality in surgical settings. Coexisting premorbid illness and postoperative complications were found to be associated with death. This study aimed to analyze various etiologies that cause peritonitis and shed light on the factors responsible for unsatisfactory results.

Method

This longitudinal study included 309 patients above 12 years of age, of either gender, with confirmed diagnosis of peritonitis. Exploratory laparotomy was done to identify the cause of peritonitis. Patients were monitored postoperatively till their discharge or death for the development of complications.

Results

Our results showed that the most common cause of acute peritonitis was duodenal perforation (26.2%), followed by typhoid ileal perforation (24.2%) and ruptured appendix (16.8%). At least one complication was observed in 31% of the participants. The most common complication was dehydration (18.8%), followed by septicemia (11.3%) and paralytic ileus (6.4%). Ten (3.2%) patients died in the hospital.

Conclusions

Acute peritonitis is a serious surgical emergency caused by a number of diseases. Early surgical treatment along with antibiotics, followed by aggressive resuscitation can yield improved outcomes in patients with peritonitis.

## Introduction

Peritonitis, an inflammation of the peritoneum, is a life-threatening acute surgical emergency. It presents with severe abdominal pain and is a significant cause of morbidity and mortality ranging from 10%-60% in surgical settings [1]. Existing literature shows that etiologies of peritonitis vary by geographic locations and local environmental factors with genetic predisposition. Appendicitis and typhoid ileal perforation are the common causes of peritonitis with an estimated prevalence of about 43.1% and 35.1%, respectively [2-3]. Other causes of peritonitis include gastroduodenal perforations, intestinal volvulus, ruptured abscesses, traumatic bowel perforation, perforated peptic ulcers, primary/idiopathic peritonitis, tubo-ovarian abscesses, and amoebic colonic perforations. Knowledge of several distinct causes and presentation of peritonitis in a particular setting will lead to improved local care and better overall understanding of the disease process, as cause is directly related to prognosis [2-3].

Aggressive fluid resuscitation and early surgical intervention are the mainstay of therapy of peritonitis. Enterocutaneous fistulas, surgical site infection, sepsis, and multiorgan failure are the commonest complications seen in surgical settings. Others include abdominal compartment syndrome, wound dehiscence, and respiratory insufficiency. Complications are influenced by advanced age and comorbidities. Coexisting premorbid illness and postoperative complications were found to be associated with death [4,5]. Despite the rapid advancement in surgical techniques, modified antimicrobial therapies, and intensive care support, the management of peritonitis continues to be more demanding, challenging, and complex than ever [4].

Severely ill patients usually present in the late stage of the disease. Consequently, there is less time for diagnostic approaches and proper decision-making for a strong treatment method. Lack of awareness, late presentation, and its correlation with morbidity and mortality demonstrate that there is room for betterment in medical treatment by a thorough examination of the etiologies, presentation, and results. Prior studies primarily focused on single etiologies; therefore, they have not focused on interconnected etiologies. The present study aimed to analyze various etiologies of peritonitis and shed light on the factors responsible for unsatisfactory results.

## Materials and methods

This longitudinal study was conducted in the emergency unit of a tertiary care hospital in Pakistan from January 2019 to March 2021. Patients with a confirmed diagnosis of peritonitis above 12 years of age and of either gender, which included 309 patients, were included in the study. Patient were enrolled via consecutive convenient non-probability technique. Their informed consent was taken. Ethical review board approval was taken before start of patient enrollment. Diagnosis of peritonitis was made based on clinical and radiological findings. Clinical findings included abdominal pain, vomiting, constipation, generalized abdominal tenderness, and absent bowel sounds. Peritonitis was indicated by X-ray showed air under the diaphragm.

Exploratory laparotomy was done to identify the cause of peritonitis. Operative findings like duodenal perforation, ileal perforation, ileal stricture with perforation, and ruptured appendix were noted in a self-structured questionnaire. Surgical intervention was done when required and the cause of perforation was treated. Patients were monitored postoperatively for the development of complications. Complications such as organ failure, septicemia, peritoneal abscess, paralytic ileus, burst abdomen, and surgical site infection were recorded. Complications due to ileostomy or any other surgical intervention were not taken into consideration in this study. Statistical Package of Social Sciences (SPSS) for Windows, version 22.0 (IBM Corp., Armonk, NY) was used to analyze data. Frequencies and percentages were calculated for categorical variables.

## Results

Acute peritonitis was more common in males (65.0%) and in the age group from 21 to 40 years (Table 1).

**Table 1:** Demographics of the participants enrolled.

Demographics	Frequency (percentage)
Gender	
Male	201 (65.0%)
Female	108 (35.0%)
Age group in years	
12–20	77 (24.9%)
21–40	97 (31.3%)
41–60	79 (25.5%)
60+	56 (18.1%)

The most common cause of acute peritonitis was duodenal perforation (26.2%), followed by typhoid ileal perforation (24.2%) and ruptured appendix (16.8%) (Table 2).

**Table 2:** Causes of acute peritonitis.

Causes	Frequency (percentage)
Duodenal perforation	81 (26.2%)
Typhoid ileal perforation	75 (24.2%)
Ruptured appendix	52 (16.8%)
Tuberculosis perforation	31 (10.0%)
Tumor perforation	21 (6.7%)
Liver cirrhosis	19 (6.1%)
Gangrenous gut	15 (4.8%)
Acute pancreatitis	06 (1.9)%
Acute diverticulitis	05 (1.6%)
Pelvic inflammatory disease	04 (1.2%)

At least one complication was observed in 97 (31.3%) participants. The most common complication was dehydration (18.8%), followed by septicemia (11.3%), and paralytic ileus (6.4%). Ten (3.2%) patients died in the hospital (Table 3).

**Table 3:** Complications of acute peritonitis.

Complications	Frequency (percentages)
No complications	212 (68.6%)
Dehydration	57 (18.4%)
Septicemia & organ failure	35 (11.3%)
Paralytic ileus	20 (6.4%)
Burst abdomen	19 (6.14%)
Surgical site infection	15 (4.8%)
Enterocutaneous fistulas	11 (3.5%)
Hepatorenal syndrome	01 (0.3%)
Hepatic encephalopathy	01 (0.3%)

## Discussion

The present study demonstrated peritonitis was significantly more prevalent in males, with a peak age group of 21-40 years. Among the factors causing peritonitis, duodenal perforation was the most common, followed by typhoid ileal perforation and ruptured appendix. Postoperative morbidity rate was 31.3% with dehydration being the most common complication, followed by septicemia and paralytic ileus. The mortality rate was 10%.

The findings of the present study are also supported by a study conducted by Choua et al. [6], who found that males were at a higher risk of developing peritonitis, and the average age of the participants was 25.8 years. The most common cause according to their study was visceral perforation, followed by diffuse appendiceal peritonitis. Participants in the present study also had duodenal perforations and affected appendix as the primary cause [6]. However, the morbidity and mortality rates in their study were lower than those of the present study. Another study carried out by Hagos et al. also showed similar results [7]. Males were seen to develop peritonitis more frequently. Acute appendicitis and perforated peptic ulcer disease were among the common causes; problems with appendix and visceral perforations were also observed in the present study. The morbidity rate was 30.8% in their study, with wound infections and sepsis being common complications, which was also observed in the present study. However, the mortality rate in the present study was 10%. Mortality rates in some other studies were 11.8% [8] and 12.63% [9]. The possible explanation for this variation in mortality rate is early diagnosis and presentation accompanied with effective surgical treatment.

*Helicobacter pylori* is the main causative agent of acute peritonitis secondary to duodenal perforation [10]. It is more common in developing countries where people do not have access to mineral water. However, boiling tap water would prevent peritonitis, and physicians are advised to diagnose and treat *H. pylori* infections with triple therapy including proton pump inhibitors (PPIs) at an early stage to avoid secondary complications [11]. Another leading cause is typhoid ileal perforation; typhoid fever is progressively being eradicated in most parts of the world but it is still endemic in India. Typhoid frequently leads to intestinal hemorrhage causing increased mortality and morbidity [12]. Moreover, the third leading cause was ruptured appendix as shown in other studies by Schietroma et al. [13] and Lin et al. [14]. However, most of these causes are due to unsanitary conditions and the use of contaminated water. The adaptation of a healthy lifestyle accompanied by early diagnosis and aggressive resuscitation, in addition to prompt surgical intervention can help avoid complications of acute peritonitis [15].

## Conclusions

Acute peritonitis, a serious surgical emergency, is caused by a number of diseases. According to the present study, *H. pylori* infections leading to duodenal perforation, typhoid ileal perforation, and ruptured appendix are the most common causes. These can be avoided by leading a hygienic lifestyle. In the case of *H. pylori* infection, PPI maintenance therapy can help avoid reinfection. Moreover, early surgical treatment along with antibiotics followed by aggressive resuscitation can yield improved outcomes for peritonitis.
